# The Emerging Roles of Viroporins in ER Stress Response and Autophagy Induction during Virus Infection

**DOI:** 10.3390/v7062749

**Published:** 2015-06-04

**Authors:** To Sing Fung, Jaume Torres, Ding Xiang Liu

**Affiliations:** School of Biological Sciences, Nanyang Technological University, 60 Nanyang Drive, Singapore 637551, Singapore; E-Mails: tsfung@ntu.edu.sg (T.S.F.); jtorres@ntu.edu.sg (J.T.)

**Keywords:** viroporin, ER stress, UPR, autophagy, apoptosis

## Abstract

Viroporins are small hydrophobic viral proteins that oligomerize to form aqueous pores on cellular membranes. Studies in recent years have demonstrated that viroporins serve important functions during virus replication and contribute to viral pathogenicity. A number of viroporins have also been shown to localize to the endoplasmic reticulum (ER) and/or its associated membranous organelles. In fact, replication of most RNA viruses is closely linked to the ER, and has been found to cause ER stress in the infected cells. On the other hand, autophagy is an evolutionarily conserved “self-eating” mechanism that is also observed in cells infected with RNA viruses. Both ER stress and autophagy are also known to modulate a wide variety of signaling pathways including pro-inflammatory and innate immune response, thereby constituting a major aspect of host-virus interactions. In this review, the potential involvement of viroporins in virus-induced ER stress and autophagy will be discussed.

## 1. Introduction

Viroporins are a family of small hydrophobic viral proteins, which oligomerize to form aqueous channels on host cellular membrane and modify its permeability. Viroporins were first identified in several RNA viruses, such as the protein 2B (P2B) in picornavirus [1] and the matrix protein 2 (M2) in influenza A virus (IAV) [2]. Later, more viroporins were shown to be encoded by other RNA viruses and some DNA viruses, such as the p7 protein in hepatitis C virus (HCV), the non-structural protein 4 (NSP4) of rotavirus and the agnoprotein in JC polyomavirus (reviewed in [3]). Based on the number of membrane-spanning domains, viroporins are classified into either Class I (with a single transmembrane domain) or Class II viroporins (with two transmembrane domains forming a helix-turn-helix hairpin motif). Within each class, the proteins are further divided into subclass A and B based on the membrane topology [3]. Some viroporins (such as the 3a protein of the severe acute respiratory syndrome coronavirus, or SARS-CoV) contain more than two transmembrane domains and are proposed to form a third class [3].

With the ability to modify membrane permeability, several viroporins have been shown to play critical functions in the entry, genome replication, assembly and release during virus infection. For example, the M2 protein of IAV forms a tetrameric proton channel, which is activated in the acidic endosomes during IAV entry [4]. Protons entering the interior of virions through the M2 channel facilitate uncoating and release of the ribonucleoprotein into the cytosol [5]. Moreover, during the maturation stage, M2 protein localizing to the trans-Golgi network increases the luminal pH, thus preventing premature conformational change of the glycoprotein hemagglutinin (HA) before the virions are transported to the plasma membrane for budding [6]. Similarly, the p7 protein of HCV has also been shown to be crucial for the assembly and release of infectious virions [7].

Apart from their direct involvement in the virus replication cycle, a number of viroporins have also been demonstrated to affect normal physiology of the host cells and contribute to the pathogenesis of viral infections. For example, viroporins from various RNA viruses are able to induce caspase-dependent apoptosis in the cultured cells [8]. On the other hand, the Viral protein U (Vpu) of human immunodeficiency virus 1 (HIV-1) may interact with cellular proteins such as cluster of differentiation 4 (CD4) and tetherin [9,10]. CD4 is the receptor for HIV-1, and the expression of CD4 on the cell surface inhibits efficient virus budding. The interaction between Vpu and CD4 retains the newly synthesized receptor in the endoplasmic reticulum (ER) and facilitates its degradation via the ER-associated degradation (ERAD) pathway [11]. Vpu also triggers the internalization and lysosomal degradation of tetherin [10], which is an interferon-induced restriction factor that may inhibit the release of HIV-1 virions [12].

Many viroporins have been shown to localize to the ER and/or its associated membrane networks (Reviewed in [3]). The presence of these channel proteins might disrupt the membrane potential and affect normal physiology of the ER (Reviewed in [13,14]). Indeed, recent studies on some viroporins have implicated their involvement in modulating the ER stress response and autophagy induction in the infected cells [15,16,17]. In this review, we will briefly highlight the signaling pathways related to ER stress response and autophagy, followed by some hypothetical mechanisms by which viroporins might regulate these pathways. A few well-characterized examples from recent published studies will then be reviewed, and several small membrane-associated viral proteins without confirmed ion channel activities will also be discussed.

## 2. Overview of the Signaling Pathways of ER Stress and Autophagy

**Figure 1:**
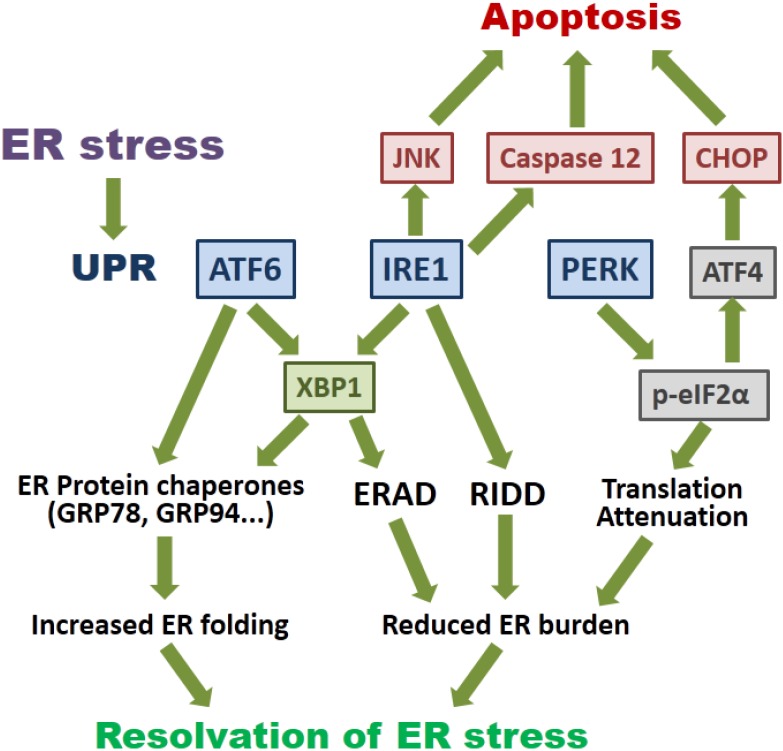
Signaling pathways of the endoplasmic reticulum (ER) stress response. ER stress induces the activation of unfolded protein response (UPR). Transcriptional activation of the transcriptional factor-6 (ATF6) induces X-box protein 1 (XBP1) and ER protein chaperones to increase the ER folding capacity. Activated inositol-requiring protein-1 (IRE1) mediates splicing of the XBP1 mRNA, and the spliced XBP1 protein enhances ER folding and the ER-associated degradation (ERAD). Non-specific activity of IRE1 also induces IRE1-dependent mRNA decay (RIDD), reducing the amount of ER-associated mRNAs. IRE1 also contributes to ER stress-induced apoptosis via c-Jun N-terminal kinase (JNK) and caspase 12. Activated PKR-like ER protein kinase (PERK) mediates the phosphorylation of eIF2α, leading to a global translation attenuation. Signaling via the activating transcription factor 4 (ATF4) and C/EBP homologous protein (CHOP) pathway also promotes apoptosis induction during prolonged ER stress. See text for detail.

In eukaryotic cells, the ER is the major site where secreted and transmembrane proteins are synthesized and folded. Depending on the physiological state and environmental conditions, the protein flux into the ER may vary substantially. When too many proteins enter the ER, unfolded proteins accumulate in the ER lumen and cause ER stress. To maintain ER homeostasis, cells have evolved signaling pathways that are collectively known as the unfolded protein response (UPR). The UPR consists of three branches of signaling pathways named after the transmembrane ER stress sensors: PKR-like ER protein kinase (PERK), activating transcriptional factor-6 (ATF6) and inositol-requiring protein-1 (IRE1) (Figure 1).

Activated PERK phosphorylates the α-subunit of eukaryotic initiation factor 2 (eIF2α), leading to global translation attenuation [18]. However, some genes could bypass this translation blockage and are preferentially expressed. These include activating transcription factor 4 (ATF4) and C/EBP homologous protein (CHOP), which are essential for the feedback regulatory loop to restore protein translation at the resolving phase of UPR [19]. CHOP also triggers apoptosis in cells under prolonged and irremediable ER stress [20,21]. Phosphorylation of IRE1 activates its RNase activity, which splices the mRNA of X-box protein 1 (XBP1) by removing a 26-nt intron [22]. The spliced XBP1 (XBP1s) translocates to the nucleus and induces the expression of UPR genes, including ER protein chaperones and components of the ERAD pathway [23]. Apart from specific splicing of the XBP1 mRNA, IRE1 has been demonstrated to mediate degradation of ER-localized mRNAs, a process known as IRE1-dependent mRNA decay (RIDD) [24]. Activated IRE1 has also been shown to induce the phosphorylation of pro-apoptotic c-Jun *N*-terminal kinase (JNK) and caspase-12 dependent apoptosis [25,26]. Lastly, ATF6 translocates from ER to the Golgi under ER stress, where it is sequentially cleaved by two proteases: S1P (site 1 protease) and S2P [27]. This cleavage releases the cytosolic domain, which translocates to the nucleus and activates genes harboring an ER stress response element (ERSE). ER protein chaperones, such as glucose regulated protein 78 kDa (GRP78), and ER-resident enzymes, such as protein disulfide isomerase (PDI), have all been shown to be induced by ATF6 [28]. The three branches of UPR do not operate independently. Rather, the tight temporal control and cross-talk among the three branches constitute an intricate signaling network [29].

**Figure 2:**
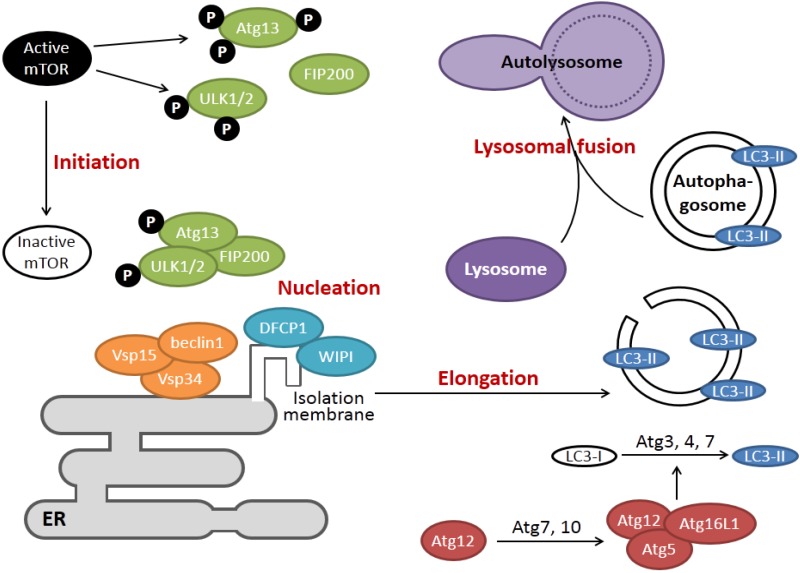
The key players in the four stages of cellular autophagy. During initiation, inactivation of the mammalian target of rapamycin (mTOR) kinase leads to hypo-phosphorylation of autophagy-related gene 13 (Atg13) and Unc-51-like kinase 1 and 2 (ULK1/2), allowing for the formation of the ULK complex. The ULK complex recruits the class III phosphatidylinositol-3-OH kinase (PI3K) complex (coiled-coil, myosin-like Bcl2 interacting protein 1 (beclin1)-vacuolar protein sorting 15 (Vsp15)-Vsp34) at the nucleation sites in the ER, where it induces the formation of isolation membrane via the activities of double FYVE domain-containing protein 1 (DFCP1) and WD-repeat protein interacting with phosphoinositides (WIPI). The elongation of isolation membrane requires the function of two ubiquitin-like systems, which results in the lipidation and conjugation of microtubule-associated proteins 1A/1B light chain 3C (LC3)-II to the autophagosome. Finally, the autophagosome fuses with lysosome to form autolysosome, and the cargos are degraded and recycled.

Macroautophagy (hereafter referred to as autophagy) is an evolutionarily conserved “self-eating” process where part of the cytoplasm and/or organelles are sequestered within a double membrane vesicle (named autophagosome), which ultimately fuses with the lysosome for bulk degradation [30]. Under basal conditions, autophagy allows cells to break down long-lived proteins and damaged organelles (such as mitochondria). Autophagy is also activated under conditions such as starvation or growth factor deprivation, so that cells can recycle amino acids and fatty acids to maintain metabolism for cell survival. Autophagy is also induced under a variety of cellular stress, such as hypoxia, reactive oxygen species, DNA damage, protein aggregates, or infection of intracellular pathogens [31]. In most scenarios, autophagy facilitates stress adaptation and promotes cell survival. However, in other settings, autophagy constitutes an alternative pathway of cell death termed autophagic cell death [32]. Complete autophagy pathway can be divided into four steps: initiation, isolated membrane nucleation, elongation and lysosomal fusion (Figure 2), and each step is tightly regulated by numerous highly conserved autophagy-related genes (ATGs).

When autophagy is induced during starvation, the mammalian target of rapamycin (mTOR) is inactivated, leading to the hypo-phosphorylation of Unc-51-like kinase 1 and 2 (ULK1/2) and Atg13. This leads to the formation of the ULK complex and its translocation from cytosol to certain domains of the ER where autophagy is initiated [33]. The ULK complex then recruits the class III phosphatidylinositol-3-OH kinase (PI3K) complex, which includes vacuolar protein sorting 34 (Vsp34), Vps15 and coiled-coil, myosin-like Bcl2 interacting protein 1 (beclin1). Vsp34 is allosterically activated by beclin1 and generates phosphotidylinositol-3-phosphate (PI3P). PI3P recruits effectors such as the double FYVE domain-containing protein 1 (DFCP1) and WD-repeat protein interacting with phosphoinositides (WIPI), facilitating the nucleation of isolated membrane structures from ER [34]. Two ubiquitin-like conjugating systems play essential roles in the elongation of autophagic vesicles. The Atg12-Atg5-Atg16 complex is present on the outside of the isolation membrane and is essential for its proper elongation. In the second system, microtubule-associated proteins 1A/1B light chain 3C (LC3) is processed and conjugated to a lipid moiety (phosphatidylethanolamine (PE)) to form LC3-II. LC3-II is stably associated with both the inner and outer membrane of autophagosomes, and its biochemical and microscopic detection has been widely used to monitor autophagy [30]. The last stage of autophagy involves fusion of the autophagosome with lysosome or late endosome for cargo degradation. Detailed mechanisms regulating vesicle fusion are not completely understood. However, the small GTPase Ras-related protein 7 (Rab7), the lysosomal associated membrane protein 2 (LAMP2) and other proteins have been proposed to mediate this process [35]. The vesicle after fusion is called autolysosome and the sequestered cargos are degraded by lysosomal enzymes to recycle biomolecules to the cytoplasm.

## 3. How Viroporin Might Modulate ER Stress and Autophagy

**Figure 3:**
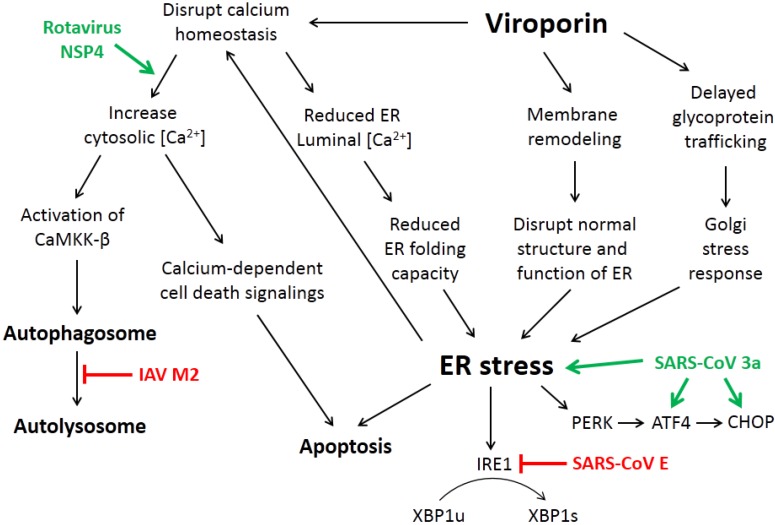
Mechanisms by which viroporins induce ER stress and autophagy. The ion channel activity of viroporins could alter the cellular calcium homeostasis, leading to a higher cytosolic [Ca^2+^], which leads to autophagy induction and activation of calcium-dependent cell death pathways. On the other hand, a lower ER luminal [Ca^2+^] reduces the ER folding capacity and leads to ER stress. ER stress induced by viroporins may also be mediated by membrane remodeling and delayed glycoprotein trafficking. The involvement of several viroporins, such as severe acute respiratory syndrome coronavirus (SARS-CoV) E and 3a protein, rotavirus NSP4 and influenza A virus (IAV) M2 protein, has been investigated recently. See text for detail.

Numerous viruses have been demonstrated to cause ER stress and induce one or more branches of the UPR in the infected cells, including but not limited to influenza virus, dengue virus, Japanese encephalitis virus, HCV and coronaviruses (reviewed in [36,37]). For envelope viruses, induction of ER stress has been mainly attributed to the production of the large receptor binding proteins, such as the HA protein of influenza virus and the spike protein of SARS-CoV [38,39]. However, several properties of the viroporins suggest that they might also modulate the virus-induced ER stress response (Figure 3).

### 3.1. ER Calcium Homeostasis

The sarco/endoplasmic reticulum Ca^2+^-ATPase (SERCA) actively transfers cytosolic calcium ion into the ER lumen, thus establishing a relatively high [Ca^2+^] in the lumen (above 100µM) and a low [Ca^2+^] in the cytosol (10–100 nM). The low cytosolic [Ca^2+^] enables a sudden release of calcium to serve as a secondary messenger of various signaling pathways, such as calcium mediated mitochondrial cell death [40]. On the other hand, the high [Ca^2+^] in the ER is essential for the function of numerous calcium dependent protein chaperones and enzymes, such as calnexin, calreticulin, GRP78 and PDI [14]. Several viroporins, including the picornavirus P2B, rotavirus NSP4 and classical swine fever virus (CSFV) p7 protein, have been shown to induce leakage of ER luminal calcium into the cytosol [1,41,42]. The perturbed ER [Ca^2+^] is likely to affect the normal activity of its protein folding machinery, thereby causing ER stress. Notably, it has also been shown that cells under ER stress induce further leakage of calcium through the ER translocon, resulting in a potential positive feedback loop that aggravates the stressed ER [43]. On the other hand, the increased cytosolic [Ca^2+^] could lead to autophagy induction, as described in a recent study for the 2B protein (P2B) of foot and mouth disease virus (FMDV) [44]. Viroporin-induced autophagy may be mediated by the activation of calcium-dependent kinases such as the calcium/calmodulin-dependent kinase kinase-β (CaMKK-β), as described below for rotavirus NSP4 (Section 4.3).

### 3.2. Membrane Rearrangement

Remodeling of intracellular membranes has been observed in cells infected with various RNA viruses [45]. For example, coronavirus infection induces the formation of double-membrane vesicles (DMVs), whereas intracellular vacuoles could be observed in picornavirus-infected cells [46,47]. These membrane structures usually co-localize with non-structural proteins and are proposed to play important roles in the replication/transcription of the viral genomes [45]. The majority of virus-induced membrane structures are derived from the ER or the Golgi network organelles, which might potentially cause ER stress [45,48]. For poliovirus, the formation of viroplasm, an ER-originated multivesicular body, has been attributed to the activity of the viroporin P2B, P2C and P2BC [49]. Moreover, when P2BC is co-expressed with P3A, membrane structures resembling those in poliovirus-infected cells can be observed [49]. However, the involvement of other viroporins in membrane remodeling has not been fully investigated. Notably, due to their morphological similarity, viral-induced DMVs have been proposed to originate from autophagosomes by some early studies in poliovirus and SARS-CoV [49,50]. However, further investigation has shown that formation of DMVs indeed utilizes mechanisms distinct from autophagy [51,52].

### 3.3. Intracellular Trafficking of Glycoproteins

Cellular and viral glycoproteins are synthesized in the ER and transported along the secretory pathway, where further folding and post-translational modifications (such as glycosylation) occur. Early studies have shown that trafficking of a model glycoprotein—the vesicular stomatitis virus glycoprotein (VSV-G)—is inhibited in cells infected with poliovirus or transfected with the P2B viroporin [53]. Similar result has been obtained for coxsackievirus, where VSV-G is arrested in the Golgi complex when co-expressed with the P2B protein, but not its mutants lacking the ion channel activity [54]. Moreover, it has also been shown that the influenza virus HA protein accumulates in the Golgi when co-expressed with the M2 protein, which is alleviated by the treatment of M2 ion channel blocker amantadine [55]. A similar delayed Golgi transport has been observed in cells treated with monensin, an ionophore that equilibrates the Golgi pH and prevents its acidification. Therefore, it is proposed that the ion channel activity of viroporins affects intracellular glycoprotein trafficking in a similar mechanism [55]. Notably, a low vesicle pH caused by the HCV p7 protein has been shown to be essential for the production of infectious virions [56]. With respect to the infected cells, accumulation of excessive proteins in the Golgi has been shown to induce Golgi stress, which is closely associated with the ER stress response and shares some of the critical signaling molecules (reviewed in [57]).

## 4. Current Studies of Viroporins Modulating ER Stress and Autophagy

In the following sections, examples of viroporins modulating the ER stress and autophagy pathways will be reviewed in detail.

### 4.1. The Coronavirus Envelope Protein Regulates ER Stress and Apoptosis

The coronavirus envelope (E) protein is very small in size (~8–10 kDa) and is present in low amounts in the virion, which explains its late discovery and recognition as a structural protein [58,59]. Most coronavirus E protein is non-glycosylated, with the exception for SARS-CoV E protein, which is partially N-linked glycosylated at Asn 66 [60]. The E protein is located in the Golgi or ER-Golgi intermediate compartment (ERGIC) in the infected cells and also in cells transfected with the E gene cDNA [61,62,63,64,65]. Although the E protein has been shown to be an integral membrane protein, its membrane topology in some cases is still under debate [60,61,66]. Results using the purified SARS-CoV E protein in micelles and SARS-CoV-infected cells [65,67], the purified MERS-CoV E protein [68] and the purified infectious bronchitis coronavirus (IBV) E protein (Torres J., Nanyang Technological University, Singapore, Biophysical studies of the infectious bronchitis coronavirus E protein, results unpublished) show only one transmembrane domain. Certain coronavirus E proteins have been shown to be palmitoylated [62,69], and this post-translational modification has been shown to be crucial for the assembly of the coronavirus Mouse Hepatitis Virus (MHV) [70].

The involvement of E protein in coronavirus assembly has been extensively investigated, which is likely mediated by its ability to induce membrane curvature and its physical interaction with the M protein [71,72]. In fact, co-expression of the M and E protein is necessary and sufficient for the formation of virus-like particles in cells [73,74]. Interestingly, the E protein is not essential for certain coronaviruses such as MHV and SARS-CoV, although deletion of the E gene results in severely crippled virions with significantly lower titers [15,75]. On the other hand, the E protein is essential for other coronaviruses such as IBV, transmissible gastroenteritis coronavirus (TGEV) and MERS-CoV. For IBV, infectious recombinant virus cannot be recovered if the E gene is deleted (Liu D.X., Nanyang Technological University, Singapore, Genetic analysis of the infectious bronchitis virus E protein, results unpublished). In terms of TGEV, deletion of E the gene results in the formation of non-infectious immature virions, which are arrested in the ERGIC and not released from the infected cells [76]. As for MERS-CoV, deletion of the E gene results in a virus that is replication-competent but propagation-defective [77].

Apart from its pivotal roles in virion assembly and morphogenesis, coronavirus E protein has also been shown to serve multiple functions and act as a viroporin in the infected cells (reviewed in [78]). The ion channel activity of coronavirus E protein has been characterized in planar lipid bilayers [79,80,81], while expression of E protein has been shown to alter membrane permeability in both bacterial and mammalian cells [62,82,83]. Biophysical studies have demonstrated that the E protein forms an ion channel that is partially inhibited by the compound hexamethylene amiloride (HMA) [80]. Recent studies have elucidated the structure of the pentameric α-helical bundle of SARS-CoV E protein ion channel [84,85], which shows voltage independent ion conductance that is also regulated by lipid charges [81]. Recombinant SARS-CoVs harboring mutations in the E protein ion channel activity display similar growth kinetics compared with the wild type virus, suggesting that the viroporin activity of E protein is not essential for SARS-CoV replication in cell culture, although it increases the fitness in competition assays [86]. However, similar viroporin mutants need to be established and characterized for other coronaviruses in the future.

Some early studies have shown that overexpression of the MHV and SARS-CoV E protein induces apoptosis in cultured cells [87,88]. The pro-apoptotic function of SARS-CoV E protein could be mediated by a reported interaction with the cellular anti-apoptotic protein B-cell lymphoma-extra-large (Bcl-xL) [88]. However, both studies adopted the cDNA transfection approach, and the function of E protein was not examined under the context of an actual infection. Moreover, addition of epitope tag has been shown to drastically affect the membrane topology and function of the IBV E protein [89]. Thus, the pro-apoptotic activity of FLAG-tag SARS-CoV E protein observed in one of the studies needs to be validated with an untagged protein [88].

A recent study utilizing a recombinant SARS-CoV that lacks the E gene (rSARS-CoV-ΔE) has demonstrated the involvement of E protein in cell stress response and apoptosis [90]. Significant splicing of XBP1 mRNA can be observed in cells infected with rSARS-CoV-ΔE but not in cells infected with wild type virus, suggesting that the SARS-CoV E protein may inhibit the activation of IRE1 during infection. Meanwhile, activation of the PERK and ATF6 branches of UPR is not significantly affected by the deletion of E. Notably, overexpression of SARS-CoV E protein is also found to down-regulate the induction of stress genes in cells treated with ER stresssors or infected with respiratory syncytial virus (RSV) [90]. The result indicates that the SARS-CoV E protein may serve as a suppressor of cellular stress response. Notably, a higher level of apoptosis is also detected in cells infected with rSARS-CoV-ΔE compared with wild type-infected cells, suggesting an anti-apoptotic function of the SARS-CoV E protein [90]. Interestingly, a previous study reported by our group has shown that the IRE1-XBP1 pathway protects cells from apoptosis induced by IBV infection [91]. In contrast, the higher level of IRE1-XBP1 activation in rSARS-CoV-ΔE-infected cells is associated with more severe cell death. It is likely that the IRE1 pathway may serve different regulatory functions in cells infected with different coronaviruses. It is also possible that the anti-apoptotic function of SARS-CoV E protein is mediated by other uncharacterized mechanisms independent of IRE1.

Another recent study by Nieto-Torres *et al.* has created recombinant SARS-CoVs incorporating mutations in the E gene that disrupt its ion conductivity [86]. Although not essential for virus replication, knockdown of E protein ion channel activity significantly reduces the fitness of SARS-CoV in competition assays and decreases the virulence in animal models [86]. Compared with wild type virus, inflammasome activation and the production of pro-inflammatory cytokines are markedly reduced in mice infected with ion channel mutants of SARS-CoV, resulting in decreased edema accumulation in the lung [86]. Since the UPR plays an important role in modulating innate immunity and pro-inflammatory response (reviewed in [37]), it is interesting to investigate the potential involvement of E protein ion channel activity in regulating UPR induction. It is also intriguing to determine whether the modulation of host stress response and the anti-apoptotic function previously characterized for SARS-CoV E protein are indeed mediated via its viroporin activity.

### 4.2. The SARS-CoV Accessory Protein 3a Induces ER Stress and Apoptosis

The 3a protein is an accessory protein of SARS-CoV with no known homolog in other coronaviruses [92]. Although the 3a protein can be detected in SARS-CoV-infected cells and the sera of SARS patients, it is not essential for virus replication in cell culture [93,94]. The protein is translated from a dicistronic subgenomic mRNA, which encodes both 3a and 3b proteins (Reviewed in [95]). In SARS-CoV-infected cells, a mixed population of transcripts with six, seven, eight and nine uridine stretches located 14 nt downstream of the initiation codon for 3a ORF have been found [96]. Among them, the hepta- and octa-uridine stretches have been shown to serve as sole signals for +1 and −1 ribosomal frameshifting, leading to the translation of 3a protein with or without the insertion of an extra amino acid at the position [96]. A number of studies have pointed to a punctate cytoplasmic localization of the SARS-CoV 3a protein, which co-localizes with the Golgi markers and may also display on the plasma membrane [93,97,98,99]. Topologically, the 3a protein contains a short extracellular *N*-terminus, three transmembrane domains, followed by a long cytosolic *C*-terminus [99,100]. Previously, Lu *et al.* [100] have shown that the SARS-CoV 3a protein forms homotetramers by disulfide bonds in the cysteine-rich domain. Moreover, when expressed in *Xenopus* oocytes, the wild type 3a protein but not its polymerization mutant demonstrates potassium ion channel activity, which is blocked by barium, a typical potassium channel blocker [100]. The same study has also shown that knockdown of 3a significantly inhibits the release of SARS-CoV, while genome replication is not affected [100].

Minakshi *et al.* [16] have explored the potential of SARS-CoV 3a protein to induce ER stress and UPR activation. Overexpression of 3a has been shown to activate luciferase reporters harboring the promoter sequences of GRP78, GRP94, ATF4 and CHOP [16]. A significant increase in the phosphorylated eIF2α is also observed in cells expressing the SARS-CoV 3a protein, although the phosphorylation status of PERK has not been determined [16]. On the other hand, transfection of 3a does not activate the IRE1 and ATF6 branches of UPR [16]. A previous study by this group has shown that activation of the PERK-eIF2α-ATF4-CHOP pathway promotes apoptosis induction during IBV infection [101]. Therefore, the ability to modulate the PERK branch of UPR could at least partially explain why overexpression of the SARS-CoV 3a protein induces apoptotic cell death in cell culture and a *Drosophila* model [102,103].

The mechanism behind 3a-induced ER stress, however, has not been investigated in detail. Similar to the M protein of some betacoronaviruses, the SARS-CoV 3a protein has been shown to acquire O-linked glycosylation post-translationally in the ER [98]. Compared with the large S protein that is massively synthesized and heavily glycosylated, the 3a protein may not significantly contribute to the increased ER burden during SARS-CoV infection. Notably, Freundt *et al.* [104] have used reverse genetics to generate a recombinant SARS-CoV with the ORF 3a deleted (Δ3a). Compared with wild type SARS-CoV, Vero cells infected the Δ3a mutant experience less membrane rearrangement and Golgi fragmentation, with a reduced amount of vesicle formation [104]. Thus, the ability of 3a to modify cellular membrane network may partially explain how it triggers ER stress, although the involvement of its ion channel activity remains unknown. Future studies will require the generation and functional characterization of recombinant SARS-CoV with the 3a ion channel activity disrupted.

### 4.3. Rotavirus NSP4 Hijacks Autophagy for Replication

Rotavirus is a non-enveloped double-stranded RNA virus that accounts for the majority of severe dehydrating diarrhea among infants and children. NSP4 of rotavirus has been shown to induce diarrhea in young mice and is the first viral enterotoxin discovered [105]. The N-terminus of NSP4 anchors the protein to the ER, whereas the *C*-terminal tail points to the cytoplasm. Due to the high plasticity of the *C-terminus*, the structure of NSP4 full protein has not been determined, although the highly conserved coiled-coil domain has been demonstrated to form a tetramer or pentamer in a pH-dependent manner [106,107]. A domain spanning amino acids 47–90 has been shown to insert into membranes and exhibit structural characteristics of a viroporin [41]. Consistently, overexpression of NSP4 increases the cytoplasmic calcium concentration, which is a well-known effect caused by rotavirus infection [41,108]. The disruption of calcium homeostasis by NSP4 is potentiated by the activation of the ER calcium sensor stromal interaction molecule 1 (STIM1), which induces store-operated calcium entry by allowing calcium influx across the plasma membrane [109]. Notably, a cleavage product of NSP4 has been found to be secreted by rotavirus-infected cells, which retains the function to perturb cellular calcium and induce diarrhea in neonatal mice [110]. On the other hand, a recent study has also shown that full-length NSP4 is secreted in a soluble oligomeric lipoprotein form, which binds to glycosaminoglycans on the surface of different cell types [111].

A recent study by Crawford *et al.* [112] has uncovered the important role of this viroporin in modulating autophagy induction during rotavirus infection. Cellular autophagy is required for efficient rotavirus replication, as production of infectious virions is markedly reduced by the autophagy inhibitor 3-methylademine, or in the Atg3−/− or Atg5−/− mouse embryonic fibroblast cells. However, autophagy is not complete in rotavirus-infected cells. Although autophagy is properly initiated (as determined by the lipidation and puncta formation of LC3), experiments using the lysosomal inhibitor Bafilomycin A1 and the tandem-fluorescent LC3 reporter suggest that rotavirus-induced autophagy does not proceed to the lysosomal fusion stage [17].

Interestingly, rotavirus NSP4 is found to co-localize with LC3 puncta in the infected cells, which then merge into larger puncta that surround the viroplasm at later stage of infection. Such co-localized NSP4/LC3 puncta can also be observed in cells overexpressing the NSP4-EGFP fusion protein, but is absent when an ion channel mutant of NSP4 is used [112]. Formation of NSP4/LC3 puncta is also significantly inhibited by the treatment of cytosolic calcium chelator or inhibitor of CaMKK-β [17]. Therefore, it is proposed that rotavirus NSP4 uses its viroporin activity to release calcium from ER to the cytosol, thereby activating CaMKK-β to induce autophagy. The virus then hijacks the autophagy pathway to deliver ER-associated viral proteins to the viroplasm, where genome replication takes place [17]. However, the detailed mechanisms of how viral proteins are specifically loaded to the NSP4/LC3-positive vesicles, and how rotavirus inhibits autophagy maturation remain to be investigated.

### 4.4. Influenza A Virus M2 Protein and the Subversion of Autophagy

As mentioned above, the M2 protein of IAV is a Class IA viroporin that forms a tetrameric proton channel, which plays essential function in the entry and maturation stage of the replication cycle. Moreover, several studies have highlighted the involvement of M2 in subverting the host autophagy pathway, and surprisingly, the ion channel activity of M2 is not required in the process.

Accumulation of autophagosomes in the perinuclear region has been observed in cells infected with several IAV strains [113]. Because the GFP-LC3 puncta do not co-localize with the lysosomal markers, it is concluded that IAV infection inhibits autophagosome maturation, which is confirmed by experiments using the lysosomal inhibitor chloroquine and the tandem-fluorescent LC3 reporter. Notably, similar blockage of autophagosome degradation can be detected in cells transfected with the M2 protein but not with other IAV proteins. Moreover, knockdown of M2 by siRNA or infection with a M2-deleted IAV mutant results in a significant reduction of autophagosome accumulation [113]. Interestingly, this activity is not shared by the homologous viroporin BM2 in influenza B virus. Importantly, treatment of M2 channel blocker amantadine or transfection of M2 viroporin mutants fails to attenuate the autophagosome accumulation phenotype, suggesting that the activity was independent of M2 ion channel activity. Notably, apoptosis induction is found higher in autophagy-deficient cells infected with IAV, suggesting that M2 may contribute to cell death by blocking the maturation step of the pro-survival autophagy pathway [113].

A recent study by Beale and Wise further characterizes the molecular mechanism by which the IAV M2 protein modulates autophagy [114]. Apart from the accumulation of autophagosomes in the perinuclear region as previously described [113], IAV infection has also been shown to re-localize LC3 to the plasma membrane in an M2-dependent manner. Moreover, sequence analysis has identified a highly conserved FVSI or FVNI motif in the cytoplasmic tail of M2. This FVxI motif matches the consensus LC3-interacting region (LIR) motif presents in other LC3-binding proteins, and is found to be essential for the M2-LC3 interaction and M2-mediated LC3 targeting to the plasma membrane [114]. Finally, the LIR of M2 is also shown to facilitate virion budding for the filamentous strain of IAV, and contribute to the virus stability at room temperature. The authors thus propose that M2-mediated subversion of autophagy may facilitate the delivery of lipid resources to the plasma membrane and promote IAV budding [114]. These studies exemplify how a viroporin could behave as a multifunctional protein and exhibit its function independent of the ion channel activity.

### 4.5. Other Small Membrane-Associated Viral Proteins Modulating ER Stress Response and Autophagy Induction without Confirmed Ion Channel Activities

Apart from the abovementioned viroporins, a number of small membrane-associated viral proteins have also been demonstrated to induce ER stress and/or autophagy, although the mechanisms of action have not been determined. While these proteins might potentially harbor ion channel activities, it is also possible that they function by interacting with known viroporins or by some distinct, unrelated mechanisms. Nonetheless, research on these small viral membrane proteins could provide insights on the functional study of viroporins, so they are also briefly reviewed here.

The family *Flaviviridae* includes several important human pathogens such as yellow fever virus, West Nile virus (WNV), dengue virus (DENV), Japanese encephalitis virus (JEV) and HCV. Flavivirus infection has been shown to induce membrane rearrangement in the ER, resulting in the formation of vesicle packets and DMVs that are associated with genome replication [115,116]. These membrane-remodeling events are driven by two hydrophobic transmembrane non-structural proteins, NS4A and NS4B [117,118,119]. The involvement of these two proteins in modulating the UPR has been documented for WNV [120]. Specifically, NS4A has been shown to activate the IRE1 and ATF6 branches of UPR, which are required for the maintenance of cell survival and innate immune evasion during WNV infection [120,121]. Overexpression of NS4B of HCV has also been shown to induce the IRE1 and ATF6 pathways, leading to the activation of NF-κB signaling [122]. In terms of autophagy, overexpression of NS4A of DENV2 has been shown to induce PI3K-dependent autophagy [123]. On the other hand, a recent study using a panel of WNV strains has shown that autophagy is induced in the WNV-infected cells except for one strain WNV-NY99, which harbors mutations in the NS4A and NS4B genes [124]. Notably, when these mutations are reverted in a recombinant virus, autophagy induction is also restored [124]. In summary, the above studies strongly support that the flavivirus NS4A and NS4B proteins modulate ER stress response and autophagy in the infected cells (reviewed in [125]), although further experiments are required to determine the mechanisms.

The non-structural protein 6 (NSP6) of coronavirus is a multi-pass transmembrane protein localized in the ER [126]. Along with two other transmembrane non-structural proteins (NSP3 and NSP4), NSP6 has been implicated to serve as a membrane anchor during the assembly of coronavirus replication complex [127]. Indeed, co-expression of SARS-CoV NSP3, NSP4 and NSP6 has been shown to induce the formation of DMVs that are similar to those observed in the infected cells [128]. Moreover, a recently identified inhibitor targeting NSP6 has been shown to inhibit DMVs formation and viral RNA replication in a broad range of coronaviruses [129]. Notably, overexpression of NSP6 alone has been shown to induce autophagosome formation in an ATG5-dependent manner [130]. Also, the ability to induce autophagy is shared by NSP6 of different coronaviruses and NSP5-7 of one arterivirus of the same order *Nidovirales* [130]. However, the autophagosomes induced by NSP6 have smaller diameters compared with those induced by starvation, suggesting that NSP6 might also inhibit the expansion of autophagosomes [131]. The mechanism behind NSP6-induced autophagy is not known, although the involvement of mTOR and the ER stress transcription factor CHOP is excluded [130]. Nonetheless, autophagy is not essential for coronavirus replication, as RNA replication and virus budding are not affected in autophagy defective cells [52,132]. However, in a recent study, short-living LC3-coated vesicles originated from the ER are shown to be hijacked by MHV for the formation of DMVs, exemplifying a novel mechanism of virus co-option of the cellular machinery [52].

## 5. Conclusions

In the recent decades, extensive structural and functional characterizations have been performed on several viroporins, revealing their importance in viral replication and pathogenesis. This is further facilitated by the identification of specific viroporin inhibitors, which have been proven to be promising antivirals for the treatment of several devastating and emerging viral infections (reviewed in [3]).

However, there has been limited investigation on the involvement of viroporins in the host response to viral infection, such as the ER stress response and autophagy. Moreover, cautions should be taken when interpreting data from some of these studies. First, phenotypes observed in cells overexpressing specific viroporins may not essentially reflect their physiological functions in the setting of an actual infection. Epitope tagging and/or forced expression of a viroporin may sometimes significantly modify its membrane topology, subcellular localization or function, leading to false-positive results. Further experiments using recombinant viruses with abolished ion channel activity for the corresponding viroporins should be used to validate these findings. Second, a certain function of a viroporin could be mediated independent of its ion channel activity. Like the abovementioned autophagy subversion activity of IAV M2 protein, the phenotype caused by a viroporin might be attributed to other domains of the protein, or its ability to interact with other viral/cellular proteins. Therefore, experiments combining virus reverse genetics and specific viroporin inhibitors are required to clarify the functional involvement of ion channel activity. Third, when comparing a recombinant viroporin mutant virus to wild type virus in terms of cellular response, it is essential to ensure that disruption of ion channel activity does not affect the replication and infectivity of the virus. Because some signaling pathways are only transiently activated during infection and highly sensitive to the infection dosage, subtle differences between the infectivity of viroporin mutant and the parental virus might lead to false-positive conclusions. Finally, apart from *in vitro* or cell-based assays, physiological significance of a viroporin needs to be validated using appropriate animal models.

Many recent studies have demonstrated the role of ER stress and autophagy in the regulation of innate immunity and pro-inflammatory response (reviewed in [133,134]). Therefore, it is hoped that research in this direction would improve our understanding of the functions of viroporins in the pathogenesis and host-virus interactions, facilitating the development of safer and more effective antivirals and vaccines.
